# Data report: transcriptome profiling of 13 tissues of Junmu No. 1 boars

**DOI:** 10.3389/fgene.2025.1641395

**Published:** 2025-09-01

**Authors:** Chunyan Bai, Jiayi Ning, Junwen Fei, Zhenbo Wang, Yu He, Jing Li, Xiaoran Zhang, Shuang Liang, Dali Wang, Hao Sun, Boxing Sun

**Affiliations:** ^1^ College of Animal Science, Jilin University, Changchun, China; ^2^ Agricultural Experiment Base of Jilin University, Changchun, China

**Keywords:** boars, RNAseq, olfactory epithelium, piglets, mRNA

## Introduction

The regulation of gene expression is influenced by multiple factors, including species origin, tissue specificity, developmental stage, and sex differences. Therefore, gene expression sequencing data obtained from various tissues of different species at different time points are of immense significance for achieving a comprehensive and clear understanding of gene functions.

**FIGURE 1:**
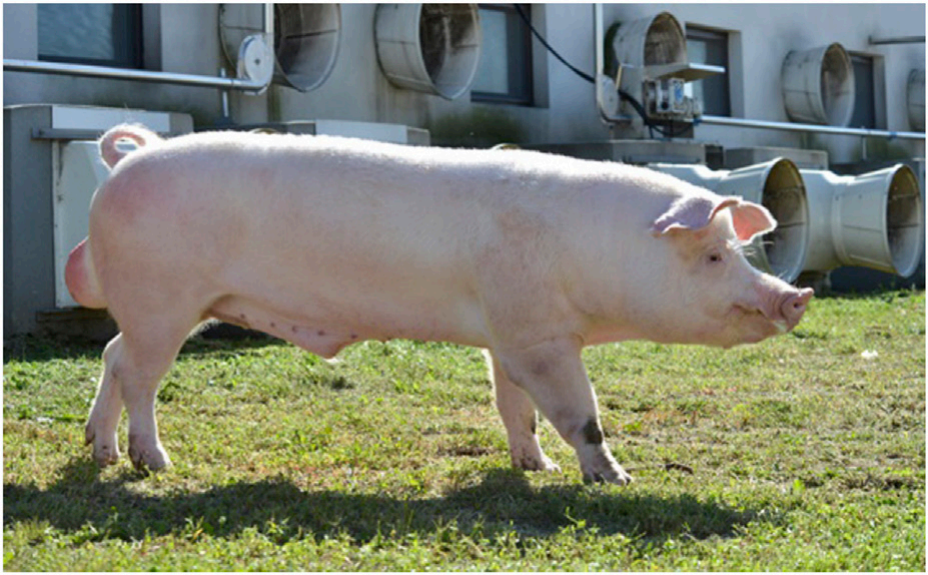
Junmu No. 1 White boar.

Numerous pig breeds exist globally. In this report, the focus is on the Junmu No. 1 White pig (Figure 1). It is a hybrid of Belgian Seghershybrid boars and Chinese Sanjiang hybrid sows, with strong artificial selection for growth traits. The Junmu No. 1 White pig is primarily utilized as a terminal sire breed, selected specifically for superior growth rate and enhanced feed conversion efficiency (Bai et al., 2017).

Generally speaking, sense of smell is closely related to its appetite, and appetite also determines the amount of feed intake, which further affects the animal’s growth rate and the farm’s economic efficiency. Meanwhile, there is limited information available on RNA-seq of olfactory tissues in pigs, despite a single recent RNA-seq survey of porcine olfactory epithelium (Yang et al., 2024) and its conspicuous absence from the Pig RNA Atlas, comprehensive transcriptomic data for this tissue remain markedly scarce, underscoring the need for the present multi-tissue dataset. Therefore, we collected RNA sequencing to profile the transcriptomes of 13 distinct tissues from growing Junmu No. 1 White pigs, with particular focus on olfactory receptor (OR) genes. By examining the relevant databases, we discovered that, for most available transcriptome data, there are few instances where so many tissues from a single pig breed have been measured simultaneously. As a result, the data we obtained is highly valuable and rich in information, and we present this data to offer valuable insights for other researchers to investigate the functions of pig genes.

## Samples collection and sequencing

The Junmu No. 1 White boars were obtained from the pig farm of Agriculture Experimental Base of Jilin University (Changchun, China). Three nursery finished healthy male piglets 70 days old with an average weight of 30 kg were randomly selected. The Spleen, ileum, brain, fat, kidney, duodenum, lung, heart, muscle, jejunum, liver, olfactory epithelium and testis tissues were collected. A small section was taken from a regular position of each tissue for each individual. Total RNA was extracted using a commercial kit (Tiangen), and sequencing was performed by Novogene Biotech (Beijing, China) using the Illumina Genome Analyzer platform.

## Data quality control and variant calling

The FASTP (Chen et al., 2018) software was used to perform quality control. The clean reads were aligned to the pig reference genome (*Sus scrofa* 11.1) using HISAT2 (Kim et al., 2019) with default parameters. Then SAM files are converted to BAM files and sorted using the Sortsam command by GATK (Van der Auwera et al., 2013). The StringTie software (Shumate et al., 2022) was used for transcript assembly and quantification. The GFFCompare software (Pertea and Pertea, 2020) was used to annotate the transcripts. Principal component analysis (PCA) was performed using DESeq2 (Love et al., 2014).

## Data description

After filtering, the average quality value of the data was significantly improved, and low-quality reads and splice sequences were effectively removed. A total of 309.8 Gb clean data was obtained. Filtered data quality values (Q30) can reach an average of 93.32%. It shows that the quality of the data has been significantly improved, providing a high-quality data base for subsequent analyses. The clean reads were aligned to the pig reference genome using HISAT2, and the results showed that the overall alignment rate was 94.61%. The results showed that most of the reads could be successfully aligned to the reference genome, indicating that the alignment process was accurate and efficient. The sequence data were deposited in the NCBI Sequence Read Achieve (SRA) and the accession number of the sequencing data was PRJNA664265. The sequencing information of each sample was shown in Supplementary Table S1.

After assembly and quantification of transcripts using StringTie, a total of 115,045 transcripts were detected with varying degrees of expression in 39 samples, these findings provided a standardised dataset for subsequent differential expression analysis. Subsequently, GFFCompare was used to annotate the transcripts, and those with a class code of “=” were specifically retained. The transcript count data obtained can be found in the figshare (https://doi.org/10.6084/m9.figshare.29192831.v1).

**FIGURE 2:**
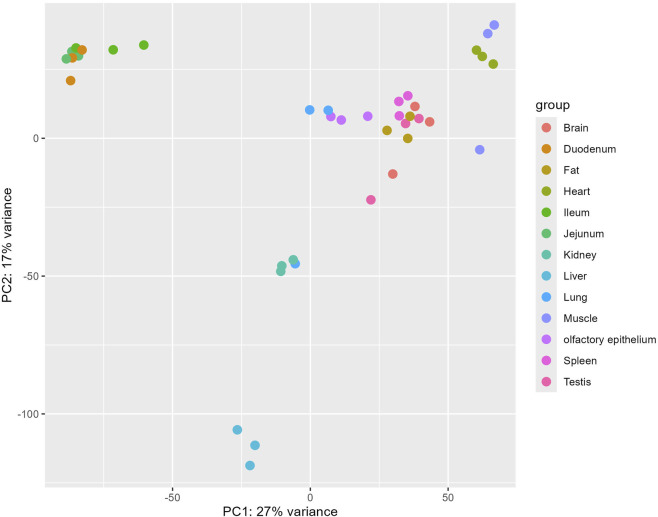
PCA plot of the 13 tissues based on gene expression count data.

To elucidate the genetic distinctions between pig olfactory epithelial tissue and other tissues for future researchers, we conducted a comparison between pig olfactory epithelial tissue and the remaining 12 tissues. The results of the principal component analysis (PCA) are depicted in Figure 2. The PCA revealed that samples from the same tissue, such as liver and muscle, clustered closely together in two-dimensional space. This clustering indicates that samples from the same tissue exhibit highly similar gene expression patterns.

In conclusion, this report provides 39 RNA-seq data from 13 tissues of three Junmu No. 1 White boars, which can provide candidate targets and molecular mechanism clues for further functional validation experiments (gene knockout, overexpression or single-cell sequencing) in the future. These datasets support analysis of gene function in porcine olfactory epithelium, advancing understanding of olfactory biology and its physiological impacts.

## Data Availability

The datasets presented in this study can be found in online repositories. The names of the repository/repositories and accession number(s) can be found below: https://www.ncbi.nlm.nih.gov/, PRJNA664265.

## References

[B1] Bai C. Pan Y. Wang D. Cai F. Yan S. Zhao Z. (2017). Genome-wide association analysis of residual feed intake in Junmu No. 1 white pigs. Anim. Genet. 48 (6), 686–690. 10.1111/age.12609 29076177

[B2] Chen S. Zhou Y. Chen Y. Gu J. (2018). fastp: an ultra-fast all-in-one FASTQ preprocessor. Bioinformatics 34 (17), i884–i890. 10.1093/bioinformatics/bty560 30423086 PMC6129281

[B3] Kim D. Paggi J. M. Park C. Bennett C. Salzberg S. L. (2019). Graph-based genome alignment and genotyping with HISAT2 and HISAT-genotype. Nat. Biotechnol. 37 (8), 907–915. 10.1038/s41587-019-0201-4 31375807 PMC7605509

[B4] Love M. I. Huber W. Anders S. (2014). Moderated estimation of fold change and dispersion for RNA-seq data with DESeq2. Genome Biol. 15 (12), 550. 10.1186/s13059-014-0550-8 25516281 PMC4302049

[B5] Pertea G. Pertea M. (2020). GFF utilities: Gffread and GffCompare. F1000Res 9, ISCB Comm J-304. 10.12688/f1000research.23297.2 32489650 PMC7222033

[B6] Shumate A. Wong B. Pertea G. Pertea M. (2022). Improved transcriptome assembly using a hybrid of long and short reads with StringTie. PLoS Comput. Biol. 18 (6), e1009730. 10.1371/journal.pcbi.1009730 35648784 PMC9191730

[B7] Van der Auwera G. A. Carneiro M. O. Hartl C. Poplin R. Del Angel G. Levy-Moonshine A. (2013). From FastQ data to high confidence variant calls: the genome analysis toolkit best practices pipeline. Curr. Protoc. Bioinforma. 43 (1110), 11.10.1–11.10.33. 10.1002/0471250953.bi1110s43 25431634 PMC4243306

[B8] Yang P. Luo T. Yang S. Zhang A. Tang Y. Chen L. (2024). Identification of olfactory receptors responding to androstenone and the key structure determinant in domestic pig. Curr. Issues Mol. Biol. 47 (1), 13. 10.3390/cimb47010013 39852128 PMC11763519

